# Microbial Heterogeneity Regulates C, N, and P Cycling Responses to Precipitation in *Casuarina equisetifolia* Forests

**DOI:** 10.3390/plants15101420

**Published:** 2026-05-07

**Authors:** Linzhi Zuo, Kaixiong Xing, Kai Wu, Ying Wang, Xiaoming Wang, Hang Zhang, Lei Li

**Affiliations:** 1 Ministry of Education Key Laboratory for Ecology of Tropical Islands, Key Laboratory of Tropical Animal and Plant Ecology of Hainan Province, College of Life Sciences, Hainan Normal University, Haikou 571158, China; zuo1914zlz@163.com (L.Z.); xingkaixiong@163.com (K.X.); zhigangzhi9@163.com (K.W.); aywying1989@126.com (Y.W.); zhanghang627@163.com (H.Z.); 2 College of Biology and Food Engineering, Guangxi Science & Technology Normal University, Laibin 546199, China; wxm081@163.com

**Keywords:** precipitation gradient, soil microorganisms, carbon, nitrogen, phosphorus cycling, functional genes, *C. equisetifolia*

## Abstract

While many environmental variables (e.g., temperature) exhibit only moderate variation, precipitation is a primary driver of soil carbon–nitrogen–phosphorus cycling in forest ecosystems, with soil microorganisms mediating these processes. Within the unique micro-ecosystems of *Casuarina equisetifolia* coastal shelterbelts, it remains unclear how microbial communities regulate carbon–nitrogen–phosphorus cycling under precipitation gradients. This study aimed to investigate the responses of microbial communities and key functional genes related to carbon–nitrogen–phosphorus cycling in *C. equisetifolia* forests on Hainan Island under varying precipitation regimes. Microbial diversity across soil layers exhibited a unimodal relationship with mean annual precipitation, with upper-layer communities more compositionally diverse than lower-layer communities. Actinobacteria, Proteobacteria, and Acidobacteria dominated the soil bacterial community and were the primary contributors to key functional genes related to soil carbon–nitrogen–phosphorus cycling, indicating that these phyla are the main functional taxa driving these elemental cycles. Microbial communities responded to precipitation changes through assemblages of precipitation-sensitive functional genes that modulated specific metabolic pathways underlying carbon, nitrogen, and phosphorus cycling. Furthermore, available phosphorus primarily drove microbial metabolic responses to precipitation changes. In conclusion, precipitation is the predominant driver of soil carbon, nitrogen, and phosphorus cycling in *C. equisetifolia* forests by directly regulating soil water content and nutrient availability. Precipitation also indirectly drives the spatial differentiation of microbial communities and selective enrichment of precipitation-sensitive functional genes across soil layers. The spatial heterogeneity of soil microorganisms, their key functional genes along the precipitation gradient and between soil layers, including Actinobacteria, Proteobacteria, and Acidobacteria, and their precipitation-sensitive key functional genes are critical regulatory machinery that orchestrate carbon, nitrogen, and phosphorus cycling across precipitation regimes and soil depths.

## 1. Introduction

Hainan Island is an important ecological region in China. Its coastline spans approximately 1580 km and is characterized by sandy coastlines, rocky shores, and mangrove forests. Located in the “typhoon corridor” of China [1], the eastern coastal zone of this island often experiences severe ecological disturbances. Heavy rainfall influences soil microbial networks’ architecture, modulated by biological soil crusts and antecedent moisture conditions [2]. Combined with soil infertility, high salinity, and water scarcity, these conditions limit the development of tall, stable, natural forests. As a result, coastal communities face varying degrees of risk to life and property [3,4,5,6]. Continuous, long-term efforts to establish coastal protection forests and improve infertile sandy soils in Hainan Province have played a crucial role in mitigating natural disasters and improving ecological conditions in coastal zones.

Hainan Island has a domed topography with a high central region that descends outward into a sequence of mountains, hills, tablelands, and plains, forming a vertically stratified, annular pattern of landforms. Mean temperatures are relatively high across the island, with warmer conditions in the southeastern coastal areas compared to the central mountainous region. Rainfall varies dramatically between the western and eastern regions, and recurrent winter–spring droughts affect the western and southern coastal areas [7,8]. These unique topographic and climatic features contribute to pronounced regional differences in vegetation diversity and growth.

*C. equisetifolia* L. is the dominant tree species used in coastal protection forests. On Hainan Island, the establishment of *C. equisetifolia* forests began in the 1950s; by 2010, they formed a nearly continuous 1528 km coastal protection belt [9]. *C. equisetifolia* has a well-developed root system, strong salinity and drought tolerance, and high nitrogen (N) fixation capacity. Therefore, *C. equisetifolia* forests are effective in windbreak and sand fixation and contribute to nutrient accumulation and soil improvement in sandy coastal areas.

Previous research has indicated that mean annual precipitation (MAP) regulates soil properties, vegetation biomass, and microbial communities’ composition and function. Microorganisms are central drivers of soil nutrient transformation and play important roles in soil respiration and biogeochemical cycling [10,11,12]. They participate in 80–90% of soil carbon (C) metabolic processes [13,14] and are strongly associated with soil N transformation [15].

The vast majority of existing coastal protection forests on Hainan Island consist of pure *C. equisetifolia* stands. Regional differences in temperature (north–south) and precipitation (west–east) have led to distinct soil physicochemical properties in these regions, influencing *C. equisetifolia* growth, biomass, and C storage. Recent studies on *C. equisetifolia* have mainly focused on germplasm resources [16,17], biomass characteristics [18], nutrient return from litter [19,20,21,22], and allelopathy [23,24,25,26,27]. However, several gaps remain in our knowledge of soil microorganisms’ responses to climatic factors in *C. equisetifolia* forests and their functional genes. To date, most studies have focused on community-level microbial composition (e.g., diversity and abundance of bacteria and fungi), but how specific functional genes involved in carbon, nitrogen, and phosphorus (C, N, P) cycling respond to precipitation gradients remains largely unknown. Furthermore, no study has systematically examined whether these functional genes exhibit vertical stratification (between soil layers) or horizontal differentiation (along precipitation gradients) in coastal *C. equisetifolia* forest soils. It is also unclear which environmental factors (e.g., precipitation, available phosphorus, soil pH, organic matter) drive changes in functional gene composition and how these genes regulate specific metabolic pathways to adapt to precipitation variation. Addressing these gaps is essential to understanding the microbial mechanisms that mediate soil nutrient cycling under changing precipitation regimes.

Based on regional differences in *C. equisetifolia* forest growth on Hainan Island, we propose two hypotheses. (1) Decreasing mean annual precipitation reduces soil nutrient availability, thereby constraining *C. equisetifolia* growth. (2) Soil microbial diversity follows a unimodal pattern along the precipitation gradient, with C, N, and P cycling processes in high- and low-precipitation sites dominated by different functional genes. Soil microorganisms adapt to regional precipitation gradients through combinations of functional genes and spatially stratified heterogeneity. To test these two hypotheses, we analyzed soils and microbial communities from *C. equisetifolia* forests along a precipitation gradient using metagenomic sequencing. We aimed to clarify how microbial communities drive changes in soil properties and reveal the mechanisms underlying C-, N-, and P-cycling microorganisms’ responses to precipitation variation and their functional genes. The results provide a foundation for future research on ecological stoichiometry within the plant–litter–soil continuum in *C. equisetifolia* forests and interactions between vegetation and microbial assembly.

## 2. Materials and Methods

### 2.1. Overview of the Study Area

**Figure 1:**
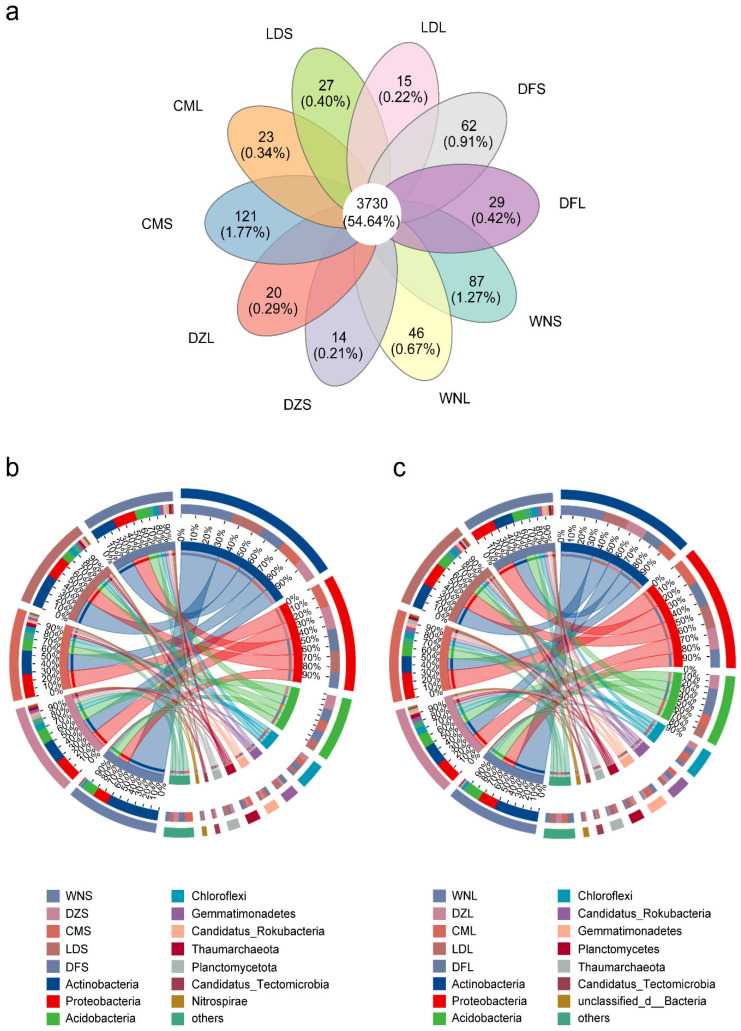
Venn diagram of the soil microbial community composition of *Casuarina equisetifolia* forests along a precipitation gradient (**a**); Circos plots of relationships between (**b**) upper soil layer samples and (**c**) lower soil layer samples with species. Samples were arranged in descending order of MAP; WNS, DZS, CMS, LDS, and DFS, denoting the upper soil layer samples of *C. equisetifolia* forests in Wanning, Danzhou, Chengmai, Ledong, and Dongfang; WNL, DZL, CML, LDL, and DFL denote the lower soil layer samples in Wanning, Danzhou, Chengmai, Ledong, and Dongfang.

Based on the MAP of various areas of Hainan Island, mature pure *C. equisetifolia* stands along the coastlines of Wanning, Danzhou, Chengmai, Ledong, and Dongfang cities were selected as sampling sites (Figure 1). The MAP gradient followed the order of Wanning > Danzhou > Chengmai > Ledong > Dongfang. Mean annual precipitation, mean annual temperature, and geographical coordinates for each sampling site are summarized in Supplementary Table S1.

### 2.2. Environmental Factor Monitoring and Sample Collection

Sample collection and experiments were conducted during the *C. equisetifolia* growing season (July to August). Within a pure *C. equisetifolia* stand, 20 × 30 m plots were selected and divided into six 10 m × 10 m subplots. In each plot, two soil temperature and moisture data loggers (MX2307, HOBO, Bourne, MA, USA) were installed away from the trees and at a depth of 20 cm away from tree roots to monitor soil water content (WC) and temperature (ST).

We employed a multi-point sampling method, selecting five sampling points within each subplot to collect soil beneath *C. equisetifolia* stands at depths of 0–5 cm and 10–20 cm (recorded as the upper and lower soil layers, respectively). For each depth, equal amounts of soil collected from the same 20 m × 30 m plot were thoroughly mixed to form a composite sample. After collection, all samples were immediately placed on dry ice and transported to our laboratory. The collected soil samples were divided into three portions, with one portion air-dried for measurement and analysis of physicochemical indicators and the other two portions stored at −80 °C for metagenomic sequencing and as a backup.

### 2.3. Measurement of Physicochemical Properties of C. equisetifolia Forest Soil Samples

For each sampling site, equal masses of samples from the same group were thoroughly mixed, and an appropriate amount of the mixed sample was separated to measure soil physicochemical indicators. Soil pH was determined using potentiometry. Organic matter (OM) and organic C (OC) content were measured using the potassium dichromate oxidation method. Total N (TN) was determined through acid digestion using the Kjeldahl method, while hydrolyzable N (HN) was measured through alkaline extraction, incubation, and volumetric analysis. Soil available phosphorus (AP) was measured through ammonium fluoride–hydrochloric acid extraction followed by the molybdenum antimony anti-colorimetric method (pH < 6.5) or sodium bicarbonate extraction followed by the molybdenum antimony anti-colorimetric method (pH ≥ 6.5). The C, N, and P content of soil microbial biomass (MBC, MBN, MBP) was separately determined using chloroform fumigation–potassium sulfate extraction–potassium dichromate oxidation, the Kjeldahl method, and the molybdenum antimony anti-colorimetric method, respectively. Litter thickness (LT) was measured on-site before sample collection. Although soil salinity is widely recognized as a key determinant of soil microbial communities, our preliminary survey indicated that it was not a primary driver of microbial community variation along the precipitation gradient under study. The five sampling sites receive high annual precipitation ranging from 949.7 to 2102.8 mm, and the predominantly sandy soil texture facilitates rapid leaching of surface salts. Likewise, pre-experimental measurements showed pH values ranging from 5.45 to 8.41 and electrical conductivity (EC) consistently below 0.4 dS/m, indicating no significant salt stress across experimental plots.

### 2.4. DNA Extraction and Metagenomic Sequencing of C. equisetifolia Forest Soil Samples

DNA was extracted from samples using the Mag-Bind^®^ Soil DNA Kit (Omega Bio-tek, Norcross, GA, USA). DNA concentration, purity, and integrity were assessed through electrophoresis on 1% agarose gel. DNA fragmentation was performed using a Covaris M220 focused ultrasonicator (Gene Company, Chai Wan, Hong Kong SAR, China), and fragments with a length of approximately 350 bp were screened to construct paired-end (PE) libraries. The library was constructed using the NEXTFLEX Rapid DNA-Seq kit (Bioo Scientific, Austin, TX, USA), and metagenomic sequencing was conducted on the Illumina NovaSeq6000 sequencing platform (Illumina, San Diego, CA, USA).

### 2.5. Data Processing and Analysis

Multidimensional statistical analyses of soil environmental factors and microbial diversity indexes were performed using the car, stats, multcomp, and emmeans packages in R software version 4.5.1 (R Core Team 2025). Linear regression analysis, correlation testing, and stratified scatter plot generation were performed using the stats (version 4.5.1), broom (version 1.0.0), ggplot2 (version 3.4.0), and ggpubr (version 0.6.0) packages in R. Random forest modeling was performed using the randomForest package (version 4.7-1.2) in R. The model was run with 1000 trees (ntree = 1000) and default mtry values. Variable importance was assessed using the percentage increase in mean squared error (%IncMSE) upon permutation. Out-of-bag (OOB) error estimation was used for internal validation, and the random seed was set to 123 to ensure reproducibility. The resulting importance scores were visualized as bar charts to rank environmental factors using the ggplot2 (version 4.0.0) and viridis (version 0.6.5) packages [28]. Circos plots of relationships between samples and species were generated in Circos-0.67-7. Schematic diagrams showing the involvement of precipitation-sensitive genes in metabolic processes were generated using the ggraph package in R. Redundancy analysis (RDA) plots and Mantel-test network heatmaps were created using the vegan package in R to analyze correlations among microbial community composition, key functional genes, and environmental factors.

## 3. Results

### 3.1. Characteristics and Importance of Environmental Factors in C. equisetifolia Forests Along a Precipitation Gradient

#### 3.1.1. Characteristics of Environmental Factors Along a Precipitation Gradient

We measured 14 environmental factors (OM, HN, AP, etc.) in *C. equisetifolia* forests distributed along a precipitation gradient. Key soil nutrient indicators (OM, OC, TN, HN, and AP) declined progressively along the decreasing precipitation gradient from Wanning (high precipitation) to Dongfang (low precipitation). In comparison, soil pH and temperature did not decline across the precipitation gradient (Table S2). Pronounced vertical stratification was consistently observed across all sampling sites, with substantially higher nutrient content (e.g., organic matter, hydrolyzable nitrogen, and available phosphorus) in the upper soil layer than in the lower soil layer. Moreover, along the decreasing precipitation gradient from Wanning (2102.8 mm) to Dongfang (949.7 mm), soil nutrient content in both layers declined, reflecting a positive correlation between mean annual precipitation and soil nutrient status. Microbial biomass and its proportion relative to total soil nutrients showed clear spatial heterogeneity along the precipitation gradient. High-precipitation sites (Wanning) tended to have lower absolute content of microbial biomass carbon (MBC) and microbial biomass phosphorus (MBP), while low-precipitation sites (Dongfang) exhibited the highest proportion of microbial biomass nitrogen (MBN) relative to total soil nitrogen (MBN/TN). In addition, the proportion of microbial biomass within total nutrients was greater in the lower than in the upper soil layers. These patterns suggest that microorganisms play an important role in soil C, N, and P transformations and exhibit strong spatial stratified heterogeneity in community structure and function (Supplementary Table S2).

#### 3.1.2. Correlation Between Environmental Factors and Soil Microbial Diversity Indices

Among the 14 measured environmental factors, seven key environmental factors (MAP, LT, WC, pH, OM, HN, and AP) were most strongly correlated with soil microbial diversity indices (Chao species richness, Shannon diversity, and Simpson dominance). Multiple comparison analyses identified MAP, AP, WC, and OM as the primary drivers of soil microbial diversity in *C. equisetifolia* forests (Supplementary Figure S1). Linear regression further showed that MAP and AP were the dominant environmental factors shaping soil microbial community structure. Both factors were negatively correlated with microbial richness and diversity but positively correlated with dominance. These effects were significantly stronger in the lower soil layer, indicating pronounced vertical stratification and region-specific regulatory patterns (Supplementary Figure S2).

### 3.2. Soil Microbial Community Composition Along the Precipitation Gradient

Metagenomic taxonomic annotation against the non-redundant protein (NR) database identified 41,269 species, 6826 genera, 2323 families, and 251 phyla across the five *C. equisetifolia* forest sites. Microbial communities differed considerably across different sampling sites. Except for Chengmai, which had the highest species richness, microbial species numbers increased with decreasing precipitation. In the upper soil layer, 6643 genera, 2291 families, and 250 phyla were identified, compared with 6437 genera, 2165 families, and 248 phyla in the lower layer. Microbial diversity was higher in the upper soil layer, consistent with our previous observation of higher nutrient levels in the upper layer (Figure 1a).

At the phylum level, Actinobacteria, Proteobacteria, and Acidobacteria dominated both soil layers. The Wanning sampling sites, which had the highest precipitation, showed the highest relative abundance of Actinobacteria and the lowest abundance of Proteobacteria, while Acidobacteria exhibited relatively stable distributions across sampling sites (Figure 1b,c).

### 3.3. Correlation Analysis Between Soil Microorganisms and Environmental Factors

**Figure 2:**
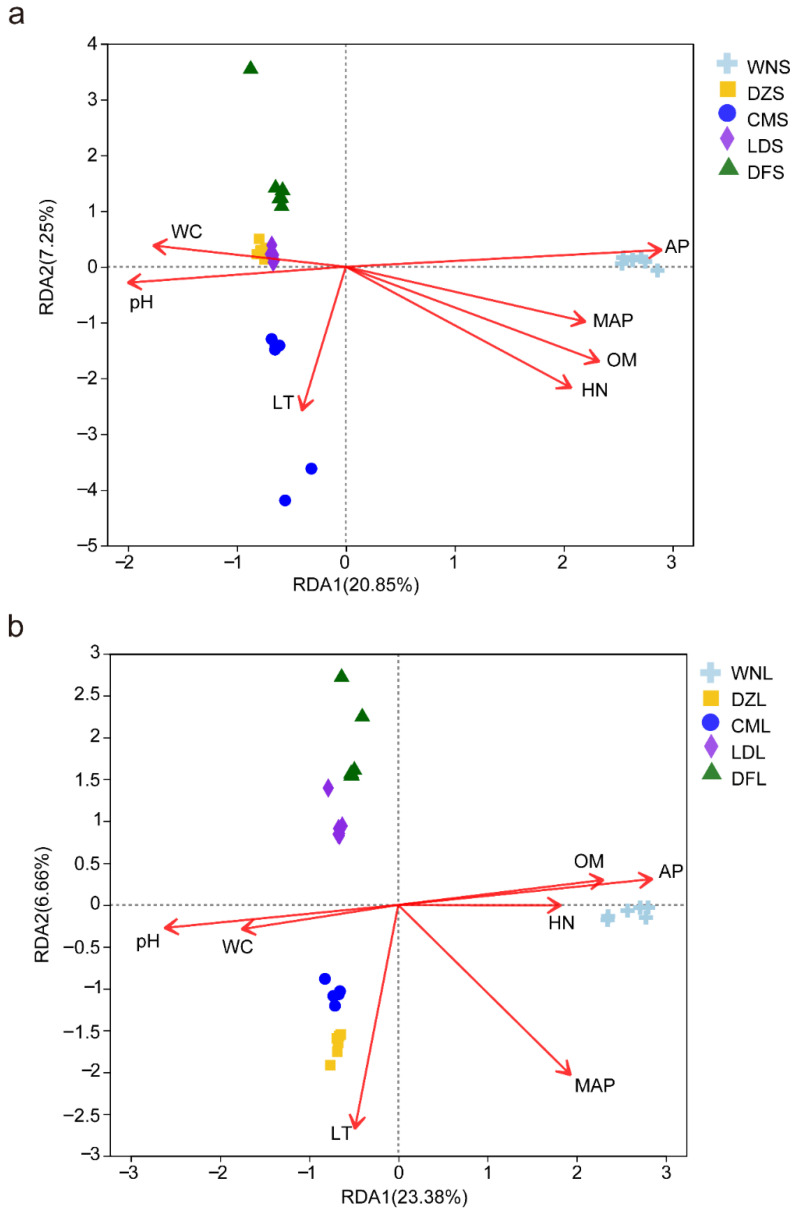
Correlations of microbial communities of the (**a**) upper soil layer and (**b**) lower soil layer of *C. equisetifolia* forests with environmental factors. Red arrows denote environmental factors, and different-colored shapes denote samples of different groups. Abbreviations: MAP = mean annual precipitation; LT = litter thickness; WC = soil water content; pH = soil pH; OM = soil organic matter; HN = hydrolyzable nitrogen; AP = soil available phosphorus.

Correlation analysis showed that key environmental factors, such as MAP, LT, WC, pH, OM, HN, and AP, significantly influenced soil microbial community structure (*p* < 0.01). However, combinations of dominant drivers differed among sampling sites and between soil layers (Figure 2).

At the sampling site scale, the dominant environmental factors at comparable soil depths differed significantly across precipitation gradients. In the upper soil layer, Wanning (high precipitation) sampling sites’ microbial community composition was most strongly correlated with AP. In contrast, communities in low-precipitation Danzhou, Ledong, and Dongfang sampling sites were mainly driven by WC and pH, whereas those at the Chengmai sampling sites correlated most strongly with LT (Figure 2a). In the lower soil layer, microorganisms at the Wanning sampling sites were primarily influenced by AP, OM, and HN. Microorganisms at the intermediate-precipitation Danzhou and Chengmai sampling sites were associated with LT, while those at the low-precipitation Ledong and Dongfang sites were primarily regulated by pH and WC (Figure 2b).

Vertically, environmental factors exhibited a significant stratification effect. Upper-layer microbial communities were mainly regulated by soil nutrient factors such as AP, HN, and OM, whereas lower-layer communities were more strongly driven by environmental input factors such as soil AP, regional precipitation, and LT. Overall, soil microbial community assembly in *C. equisetifolia* forests was jointly shaped by precipitation patterns and soil nutrients, with dominant factors shifting along the precipitation gradient and with soil depth.

### 3.4. Differential Analysis of C, N, and P Cycling-Related Functional Genes and Functional Microorganisms in C. equisetifolia Forest Soils

#### 3.4.1. Distribution of Key Functional Genes Across Samples

Our results indicate that soil microorganisms in *C. equisetifolia* forests are regulated not only by precipitation but also by the availability of soil C, N, and P. Using Kyoto Encyclopedia of Genes and Genomes (KEGG) annotations, we screened 334, 40, and 550 functional gene categories related to C, N, and P cycling in *C. equisetifolia* forests (KEGG Orthology (KO) functional hierarchies). Precipitation-sensitive genes (*p* < 0.05) were further subjected to random forest analysis and ranked by importance according to KEGG nomenclature. The top five functional genes in each nutrient cycle were selected for subsequent analyses. Functional gene distribution exhibited pronounced spatial heterogeneity across the precipitation gradient. The same metabolic pathway was governed by different functional genes across sampling sites and soil layers, forming a vertically stratified metabolic strategy (Supplementary Figure S3).

**Figure 3:**
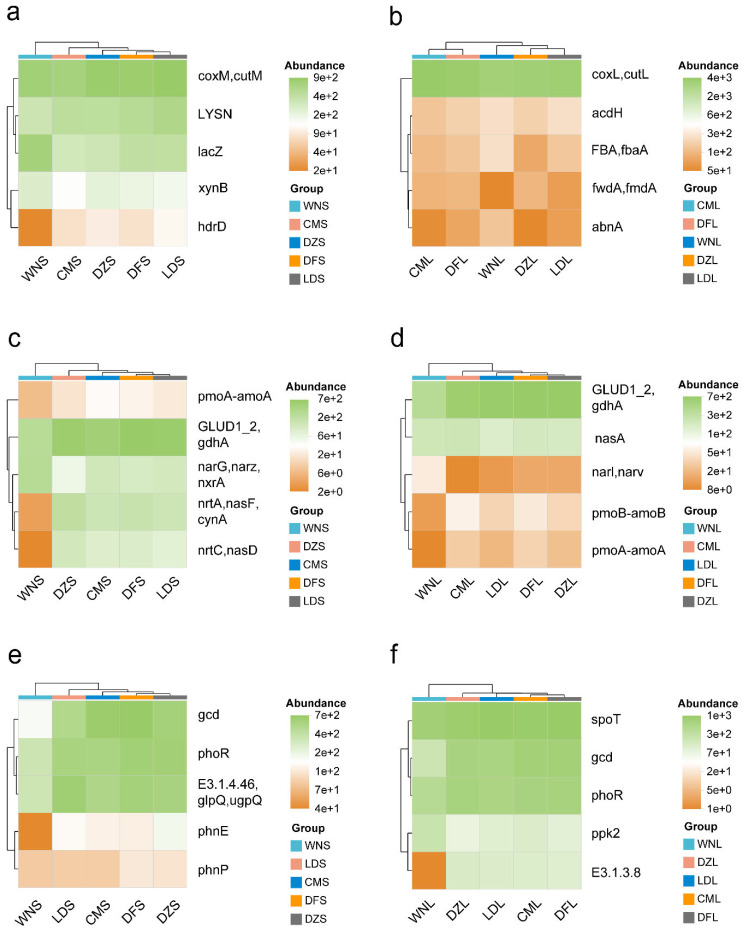
Distribution of the top five precipitation-sensitive functional genes across samples (**a**–**f**).

A comparison of key functional gene abundance in different samples revealed that C-, N-, and P-cycling genes were widely distributed across sampling sites but responded differently to the precipitation gradient. For soil C cycling, clear functional substitution occurred among sampling sites. At high-precipitation Wanning sampling sites, the decomposition-related gene (*lacZ*) was enriched in the upper soil layer, whereas C transformation-related genes (e.g., *coxM*, *cutM*, and *LYSN*) were enriched at the low-precipitation Dongfang sampling sites. Functional genes showed complementary vertical distributions as well; hydrolysis- and synthesis-related genes dominated the upper layer (e.g., *coxM*, *cutM*, and *LYSN*), while reduction- and oxidation-related genes controlled the lower layer (e.g., *coxL*, *cutL*, and *acdH*) (Figure 3a,b).

N-cycling genes exhibited polarized responses to precipitation gradients. High-precipitation Wanning sampling sites promoted nitrification-related genes (e.g., *narG*, *narZ*, and *nxrA*) but inhibited multiple genes responsible for N assimilation and ammonia oxidation (*GLUD1_2*, *gdhA*, *pmoA-amoA*, *nrtA*, *nasF*, and *cynA*). In contrast, low-precipitation Dongfang soils significantly enriched N-assimilation-related functional genes (*GLUD1_2* and *gdhA*). Strong vertical differentiation was also observed; assimilation- and nitrification-related genes dominated the upper layer, and specific reduction genes (e.g., *narI* and *narV*) dominated the lower layer (Figure 3c,d).

The distribution of genes related to P cycling also showed inverse precipitation responses. At high-precipitation Wanning sampling sites, specific P regulation and storage-related gene *ppk2* was enriched, whereas P absorption and mineralization genes (*gcd*, *phoR*, and *phnP*) were enriched at low-precipitation Dongfang sites. Vertically, upper- layer soils were dominated by genes involved in P activation and turnover (*gcd*, *phoR*, *E3.1.4.46*, *glpQ*, and *ugpQ*), while core P homeostasis genes (*spoT*, *gcd*, and *phoR*) were dominant in the lower-layer samples. These findings indicate that microorganisms adopt a multi-level adaptation strategy to precipitation variation through combinations of C-, N-, and P-cycling genes across sampling sites and soil depths (Figure 3e,f).

#### 3.4.2. Microbial Contributions of Key Functional Genes

**Figure 4:**
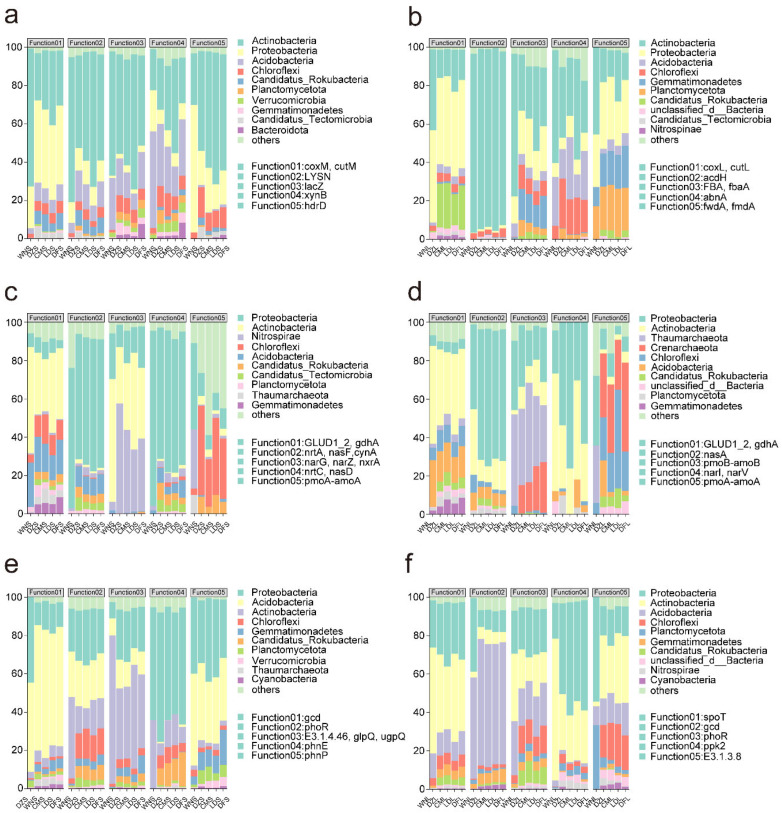
Comparison of microbial contributions to the top five functional genes along the precipitation gradient (**a**–**f**).

Analysis of species and functional contributions indicated that Actinobacteria, Proteobacteria, Acidobacteria, and Chloroflexi were the primary contributors of C-, N-, and P-cycling-related functional genes, with clear functional differentiation between soil layers. C-cycling genes exhibited complementarity vertical patterns; in the upper layer, *LYSN*, *lacZ*, and *hdrD* were dominated by Actinobacteria, and in the lower layer *acdH*, *FBA*, *fbaA*, and *abnA* were also primarily associated with Actinobacteria. In contrast, *coxM* and *cutM* in the upper soil layer and *coxL* and *cutL* in the lower layer were mainly dominated by Proteobacteria (Figure 4a,b).

N-cycling genes also showed strong taxonomic stratification. In the upper soil layer, *narG*, *narZ*, and *nxrA* were primarily contributed by Nitrospirae, while *pmoA-amoA* was dominated by Chloroflexi. In contrast, ammonia-oxidation-related genes in the lower layer, such as *pmoB-amoB*, were mainly contributed by Thaumarchaeota and Crenarchaeota (Figure 4c,d).

Microorganisms involved in P cycling exhibited a similar division. Proteobacteria dominated P transport genes (*phnE* and *spot*), Acidobacteria contributed primarily to P absorption genes (*gcd* and *phnP*), Actinobacteria were associated with multiple phosphatase genes, and Chloroflexi consistently contributed to the *phoR* gene across soil layers. Collectively, these patterns indicate that soil microorganisms establish a multi-level metabolic strategy across the precipitation gradient through systematic taxonomic allocation of specific functional genes (Figure 4e,f).

### 3.5. C, N, and P Metabolism Processes Associated with Key Functional Genes

**Figure 5:**
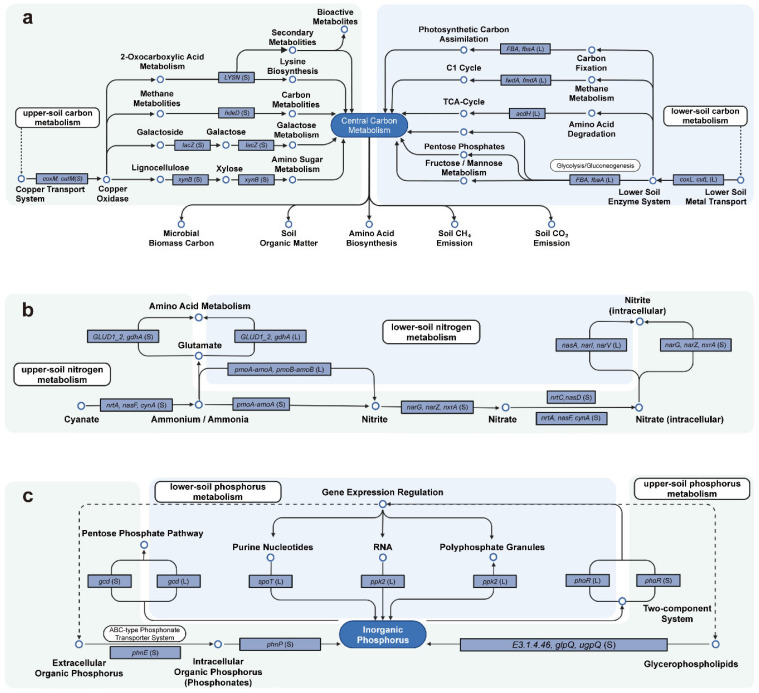
Reaction pathways in which precipitation-sensitive genes were primarily involved during (**a**) C metabolism, (**b**) N metabolism, and (**c**) P metabolism. S indicates that the gene originated from the upper soil layer, and L indicates that the gene originated from the lower soil layer.

Precipitation-sensitive key functional genes involved in C, N, and P cycling showed strong spatial heterogeneity, with clear vertical differentiation in their metabolic roles. For C cycling, the upper soil layer adapted to precipitation changes mainly through the degradation of complex OM. *xynB* and *lacZ* contributed to hemicellulose and galactose decomposition, *LYSN* regulated amino acid biosynthesis, and *hdrD* participated in methane metabolism. The lower soil layer was dominated by central C metabolism and amino acid metabolism, with representative genes including *FBA*, *fbaA*, *fwdA*, *fmdA*, and *acdH*. Metal-cofactor genes also exhibited stratified differences; *coxM* and *cutM* were active in the upper layer, whereas *coxL* and *cutL* were concentrated in the lower layer (Figure 5a).

For N cycling, the upper layer responded to changes in precipitation through the joint action of multiple N transformation pathways. This was primarily manifested as cyanate degradation (*nrtA*, *nasF*, and *cynA*), ammonia oxidation (*pmoA-amoA*), nitrite oxidation and dissimilatory nitrate reduction (*narG*, *narZ*, and *nxrA*), and ammonia assimilation (*GLUD1_2* and *gdhA*). In contrast, lower-layer responses were focused on core processes such as ammonia oxidation (*pmoA-amoA* and *pmoB-amoB*), dissimilatory nitrate reduction (*narI* and *narV*), and assimilatory nitrate reduction (*nasA*) (Figure 5b).

For P cycling, the upper layer primarily responded through the phosphonate pathway (*phnE*, *phnP*) and the glycerophospholipid pathway (*E3.1.4.46*, *glpQ*, *ugpQ*). The lower layer mainly relied on the inositol phosphate pathway (*E3.1.3.8*) and the nucleic acid degradation pathway (*ppk2*, *spot*). In addition, *gcd* and *phoR* contributed to precipitation adaptation in both the upper and lower soil layers (Figure 5c).

Collectively, microbes in *C. equisetifolia* forest soils construct functionally complementary C-, N-, and P-cycling networks across upper and lower soil layers, forming a multi-level, spatially heterogeneous response to adapt to precipitation gradients. This reflects soil microbial communities’ functional flexibility and ecological niche differentiation.

### 3.6. Correlation Analysis Between Key Functional Genes and Environmental Factors

**Figure 6:**
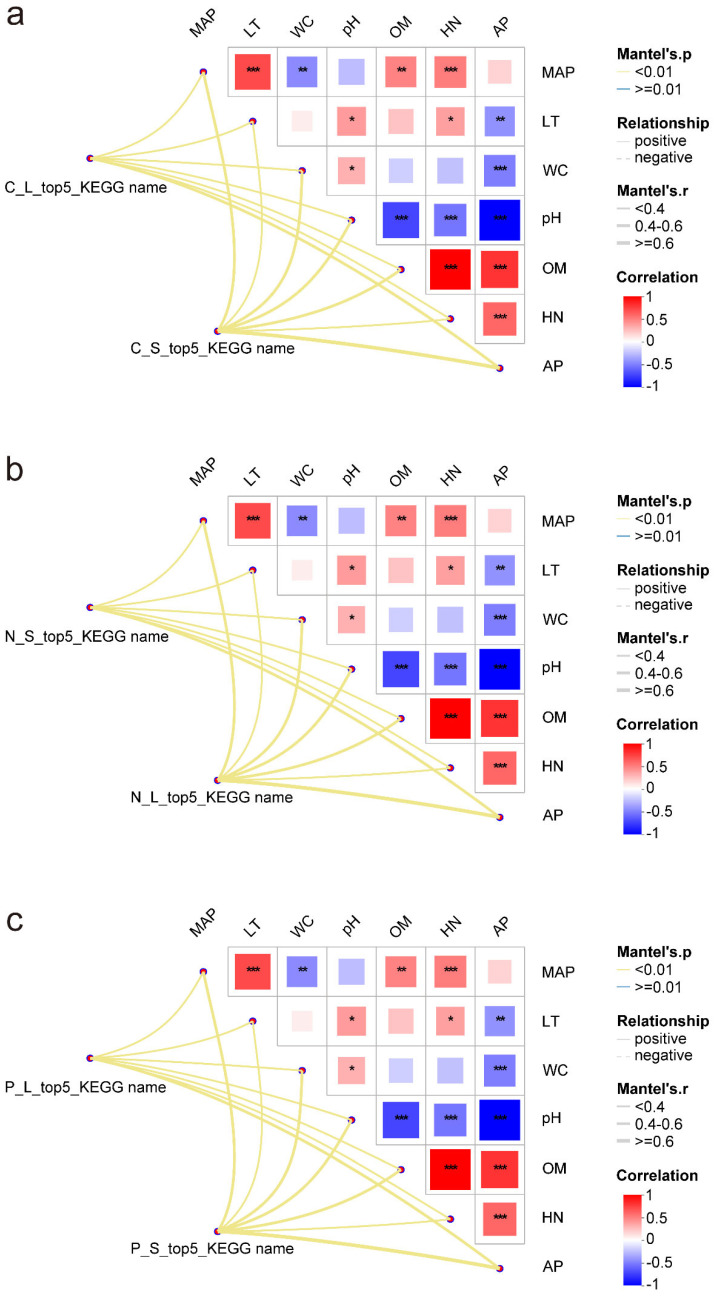
Correlation analysis between (**a**) C cycling-, (**b**) N cycling-, and (**c**) P-cycling-related functional genes sensitive to precipitation and environmental factors. Different colors in the heatmap reflect positive or negative correlations, and the color intensity corresponds to the magnitude of the correlation. Asterisks within the cells denote significance levels: * for 0.01 < *p* ≤ 0.05, ** for 0.001 < *p* ≤ 0.01, and *** for *p* ≤ 0.001.

Correlation analysis between precipitation-sensitive C-, N-, and P-cycling functional genes and major environmental factors revealed significant associations with seven key environmental factors, including MAP (*p* < 0.01). C-cycling genes were mainly regulated by AP and OM and exhibited strong spatial heterogeneity; upper-layer genes responded more strongly to pH, while lower-layer genes were more sensitive to MAP (Figure 6a). N cycle gene responses were driven primarily by AP, OM, and HN, with similar patterns in both soil layers (Figure 6b). For P-cycling genes, AP, pH, and OM were the dominant factors in both layers (Figure 6c). Overall, AP and OM were the core environmental drivers of microbial metabolic responses to precipitation changes. pH and MAP exerted layer-specific regulatory effects on different metabolic pathways, indicating that microbial communities in different soil layers employ distinct strategies to cope with moisture variability.

## 4. Discussion

### 4.1. Patterns of Change in Soil Microorganisms of C. equisetifolia Forests Along a Precipitation Gradient

Analysis of soil microbial community composition in *C. equisetifolia* sampling sites showed that with the exception of Chengmai sampling sites, microbial diversity decreased with increasing precipitation (Figure 1a). This contrasts with the work of Zhai et al. in temperate grasslands, where decreased precipitation lowered bacterial and fungal diversity but increased soil microbiome network complexity [29]. Several factors may explain this discrepancy. First, grassland soils harbor more drought-adapted microbial taxa and can exhibit compensatory responses (e.g., increased microbial biomass) under moderate precipitation reduction, whereas forest soil microbes—including those in our study—are more sensitive to water stress. Second, bacterial diversity is generally less responsive to precipitation changes than fungal diversity. Bacterial diversity remains stable across various precipitation regimes, while fungal diversity declines significantly only under extreme or short-term drought. Zhai et al. likely observed declines in both fungal and bacterial diversity in temperate grasslands because drought intensity or duration in their study reached the threshold required to affect bacterial communities. In contrast, the precipitation gradient in our study area (Hainan Island) may not have crossed this bacterial threshold; therefore, the observed changes in microbial diversity are likely driven primarily by fungal communities. Third, differences between forests and grasslands in precipitation intensity, duration, and background climate may also contribute. Hu et al. demonstrated that the magnitude and duration of precipitation change are critical mediators of microbial responses and that microbial biomass responses to drought are further modulated by factors such as mean annual temperature and elevation [30]. With mean annual temperature showing limited variation, precipitation is a key determinant of soil microbial community composition. However, the direction and magnitude of its influence depend on ecosystem type and site conditions.

The Chengmai sampling site, located at an intermediate position along the precipitation gradient (Table S1), had the highest number of soil microbial species. This indicates that moderate precipitation—approximately 1500 mm per year—provides optimal conditions for soil microbial survival in *C*. *equisetifolia* forests. Plant litter is an essential source of soil microorganisms and nutrients, and precipitation influences the degree of litter decomposition, soil water content, and the rate at which microorganisms and nutrients are transported downward [30]. At the Chengmai site, this moderate precipitation regime promotes adequate litter decomposition, maintains favorable soil moisture, and facilitates balanced downward microorganism and nutrient transport. Therefore, for *C. equisetifolia* forests, a mean annual precipitation of approximately 1500 mm represents an optimal precipitation regime that enhances soil microbial diversity.

Our results revealed that microbial diversity was consistently higher in the upper soil layer than in the lower layer across all five sampling sites along the precipitation gradient (Wanning, Danzhou, Chengmai, Ledong, and Dongfang; Table S1), consistent with the vertical variation in soil nutrient content between the upper and lower soil layers (Figure 1a; Supplementary Table S2). Similarities in microbial community composition between layers suggest that upper soil may function as the primary source pool for soil microorganisms in deeper layers, with downward transfer occurring through processes such as leaching (Figure 1b,c). The upper soil layers at Dongfang and Chengmai (low to intermediate precipitation) contained the highest numbers of microbial genera, while Danzhou and Wanning (high precipitation) had the lowest richness across soil depths (Figure 1a).

Analysis of soil physicochemical properties revealed no significant differences in carbon-, nitrogen-, and phosphorus-related indicators between the upper and lower soil layers in Dongfang (Supplementary Table S2). This may be attributed to low levels of precipitation at this site, limiting nutrient leaching and translocation between soil layers and resulting in vertically homogeneous nutrient conditions. Despite these lower levels of precipitation, Dongfang exhibited relatively high microbial diversity, suggesting that stable soil physicochemical environments may support a wider range of microbial taxa. Similarly, Arunrat et al. found that continuously fallow soils (undisturbed for seven years) were dominated by oligotrophic taxa adapted to low-nutrient conditions, relying on symbiotic or energy-efficient nutrient acquisition strategies. In these communities, genes involved in energy production, carbohydrate metabolism, and sulfur oxidation were also enriched, reflecting broader metabolic capabilities under stable conditions [31]. Their findings indicate that land use history shapes distinct microbial assemblages and metabolic potential, with microorganisms adapting to more stable but nutrient-limited environments. This pattern aligns with our observations.

### 4.2. Patterns of Functional Gene Responses to Precipitation Change

Precipitation-sensitive functional genes also showed clear spatial heterogeneity across sites and soil layers (Figure 3 and Figure 4). Differences in functional gene abundance indicate that microorganisms adapt to precipitation-induced environmental shifts by constructing functionally complementary C, N, and P metabolic networks across different soil layers (Figure 5). Distinct combinations of key genes are regulatory factors contributing to the establishment of spatially complementary (i.e., layer-specific and site-specific) adaptive strategies.

Across all sites, Actinobacteria, Proteobacteria, Acidobacteria, and Chloroflexi were primary contributors to precipitation-responsive C-, N-, and P-cycling genes (Figure 1b,c and Figure 4). They were also dominant taxonomic groups in the overall soil microbial community. Previous studies have reported that these phyla are major functional microorganisms, and they are commonly used as bioindicators of land use type succession [32]. For instance, Actinobacteria are widely distributed in natural ecosystems, such as soil, rhizosphere, and vegetation, and they produce metabolites with key roles in physiological processes, such as plant growth [33,34,35]. Proteobacteria include typical nitrogen-fixing taxa and are commonly distributed in legumes and other nitrogen-fixing plants [36,37]. Proteobacterial communities participate in bioremediation and heavy-metal-contaminated soil degradation, as well as pollutant transformation, such as antibiotics in water [38,39,40]. Acidobacteria, one of the major bacterial phyla commonly found in soil, degrade plant-derived polymers, such as cellulose, hemicellulose, and xylan, and contribute to sulfur cycling [41,42,43,44,45,46]. Abundances of Proteobacteria and Acidobacteria are shaped by soil pH and nutrient availability [33,47,48].

### 4.3. Correlations Among Microorganisms, Functional Genes, and Important Environmental Factors

Correlation analyses revealed that soil microbial community assembly in *C. equisetifolia* forests is jointly controlled by climatic–edaphic variables (precipitation, soil WC, and pH) and soil nutrients (available phosphorus, OM, and HN). In high-precipitation sites, soil nutrients were predominant drivers, whereas in low-precipitation sites climatic–edaphic factors exerted the strongest influence. Along the soil profile, microbial communities in the upper layer were governed primarily by soil nutrients, whereas those in the lower layer relied on the combined effects of climatic and edaphic variables and soil nutrients (Figure 2). For precipitation-sensitive functional genes, soil available phosphorus and OM were key factors mediating gene responses to precipitation change; genes in the upper layer were additionally regulated by pH, whereas those in the lower layer were further influenced by precipitation amount (Figure 6). Collectively, these findings indicate that environmental control over soil microorganisms and their functional genes exhibits spatial heterogeneity across both precipitation gradients and soil depths.

Previous research has reported that soil pH is primarily influenced by factors such as WC, OM, and the accumulation of N and P. WC is a key limiting factor for vegetation growth, and precipitation is its primary source; it also affects N availability in semi-arid soils [49,50]. Zhou et al. showed that WC is significantly lower in plantations than in natural forests [51]. Our findings indicate that precipitation is the dominant factor governing nutrient accumulation and microbial diversity in *C. equisetifolia* coastal protection forests on Hainan Island. Furthermore, we found that P emerged as a key regulator of soil microbial communities and functions. While others, such as Tang et al., have reported weaker effects of P and sulfur on soil microbial community succession compared to C and N [52], our results indicate that P is a central driver of microbial growth, division, metabolism, and community structure in this system, possibly because of its fundamental role in nucleic acids, phospholipids, and ATP. Therefore, our study emphasizes that together with precipitation, phosphorus (P) availability acts as a key regulator of soil microbial communities and carbon cycling in *C. equisetifolia* forests. This may be related to phosphorus limitation in the soil, which is commonly induced by the high-temperature and high-humidity environment of *Casuarina* plantations in Hainan.

### 4.4. Limitations and Perspectives

Soil microbial communities were characterized at only two depths (0–5 cm and 10–20 cm). While our results demonstrate significant vertical stratification of microbial diversity, community composition, and functional gene distribution between these two functionally distinct horizons, this sampling resolution is insufficient to fully capture the continuous pattern of spatial heterogeneity across the entire 0–20 cm soil profile. Spatial heterogeneity operates at multiple scales, and a two-depth comparison, although informative, provides only a coarse view of the vertical dimension. Future studies should employ a higher-resolution depth sampling strategy (e.g., 5 cm intervals or finer) to more comprehensively examine vertical gradients in microbial communities and their functional potentials. Such high-resolution data would also allow for more robust statistical modeling of the relationships between depth, soil properties, and microbial responses to environmental perturbations.

## 5. Conclusions

In this study, we used metagenomic methods to characterize *C. equisetifolia* forest soils’ physicochemical properties, microbial communities, and functional genes along a precipitation gradient. We analyzed the relationships between microbial community composition, key functional genes, and major environmental factors and identified C-, N-, and P-cycling functional genes sensitive to precipitation and their primary metabolic pathways. As MAP declined, soil nutrient concentrations decreased across both horizons, with consistently higher levels in upper soil. In contrast, microbial diversity exhibited a unimodal response, increasing initially and then decreasing along the precipitation gradient. Soil microbial community composition across sampling sites was primarily shaped by MAP, AP, WC, and OM. Patterns of microbial diversity followed the same gradient as soil physicochemical properties, with the greatest diversity occurring at intermediate precipitation sites. Actinobacteria, Proteobacteria, and Acidobacteria dominated among soil microorganisms and contributed most to key functional genes. At the gene level, microbial responses to precipitation changes were spatially differentiated: along the precipitation gradient and across soil depths, microbial communities assemble distinct combinations of functional genes, adapting to changes in environmental factors such as precipitation by regulating corresponding metabolic pathways. High-precipitation sites were mainly enriched in carbon decomposition genes, nitrification process genes, and phosphorus regulation and storage genes, whereas low-precipitation sites were mainly enriched in carbon transformation genes, nitrogen assimilation genes, and phosphorus absorption and mineralization genes. For C cycling, *xynB* and *LYSN* in the upper soil layer primarily governed the degradation of complex OM, such as hemicellulose, while *FBA* and *fbaA* in the lower layer were associated with central C and amino acid metabolism. For N cycling, *GLUD1_2*, *gdhA*, *nrtA*, *nasF*, and *cynA* dominated ammonia oxidation and cyanate degradation in the upper layer, whereas *narI*, *narV*, and *nasA* regulated nitrate reduction in the lower layer. For P cycling, *phnE* and *phnP* controlled the phosphonate pathway in the upper layer and *ppk2* and *spoT* predominated in nucleic-acid-related pathways in the lower layer. Overall, our results suggest that precipitation influences nutrient sequestration and the assembly of microbial communities and functional genes in *C. equisetifolia* forest soils, acting directly or indirectly through factors such as WC and pH. Precipitation is a critical environmental driver that must be considered in *C. equisetifolia* forest soil micro-ecosystems. Precipitation amount primarily drove soil C, N, and P cycling in *C. equisetifolia* coastal forests, with the spatial heterogeneity of soil microorganisms and their key functional genes acting as critical regulatory machinery. By linking microbial community structure to biogeochemical function, our study provides insights for sustainable *C. equisetifolia* shelterbelt management and soil nutrient improvement.

## Data Availability

Data are available from the NCBI database (https://www.ncbi.nlm.nih.gov/; accession number: PRJNA1441116; accessed on 22 March 2026).

## Supplementary Materials

The following supporting information can be downloaded at https://www.mdpi.com/article/10.3390/plants15101420/s1, Figure S1: Variable importance plot based on random forest analysis ranking environmental factors by their contribution to variations in soil microbial diversity indices (Chao, Shannon, and Simpson indices); Figure S2: Correlation between environmental factors and soil microbial diversity indices in *C. equisetifolia* forest soils along a precipitation gradient; Figure S3: Ranking of key functional genes (KEGG name level) by their relative importance in *C. equisetifolia* forest soils along a precipitation gradient; Table S1: Geographic coordinates and climatic information of *Casuarina equisetifolia* sampling sites along a precipitation gradient on Hainan Island, China; Table S2: Physicochemical properties of *C. equisetifolia* forest soils along a precipitation gradient, Hainan Island; Table S3: Microbial biomass characteristics of *C. equisetifolia* forest soils along a precipitation gradient.

## Abbreviations

The following abbreviations are used in this manuscript:

MAP	Mean Annual Precipitation
MAAT	Mean Annual Air Temperature
LT	Litter Thickness
WC	Soil Water Content
ST	Soil Temperature
pH	Soil pH
OM	Soil Organic Matter
OC	Soil Organic Carbon
TN	Total Nitrogen
HN	Hydrolyzable Nitrogen
AP	Available Phosphorus
MBC	Microbial Biomass Carbon
MBN	Microbial Biomass Nitrogen
MBP	Microbial Biomass Phosphorus
