# Supplementary material for: Microbial Heterogeneity Regulates C, N, and P Cycling Responses to Precipitation in Casuarina equisetifolia Forests
Source: Plants (Basel). 2026 May 7;15(10):1420. doi: 10.3390/plants15101420 (PMC13210579; doi:10.3390/plants15101420)
Supplement: Supplementary file 1 [file plants-15-01420-s001.zip › plants-4249105-supplementary.pdf]

Supplementary Materials

Microbial heterogeneity regulates C, N, and P cycling responses to precipitation in *Casuarina equisetifolia* forests

Linzhi Zuo[1,2], Kaixiong Xing[1], Kai Wu[1], Ying Wang[1], Xiaoming Wang[2], Hang Zhang[1] and Lei Li[1*]

[1]Ministry of Education Key Laboratory for Ecology of Tropical Islands, Key Laboratory of Tropical Animal and Plant Ecology of Hainan Province, College of Life Sciences, Hainan Normal University, Haikou 571158, China.

[2] College of Biology and Food Engineering, Guangxi Science & Technology Normal University, Laibin 546199, China.

*Corresponding author: Lei Li, E-mail: lei-li@126.com

Table S1-S3

Figure S1-S3

Table S1. Geographic coordinates and climatic information of *Casuarina equisetifolia* sampling sites along a precipitation gradient in Hainan Island, China.

Site No.	Site location	Mean annual precipitation (mm)	Mean annual temperature (°C)	Coordinates
1	Wanning (WN)	2102.8	25	19°02′08″N，110°32′07″E
2	Danzhou (DZ)	1828.8	23.8	19°51′20″N，109°25′33″E
3	Chengmai (CM)	1512.8	24.1	19°56′32″N，109°58′59″E
4	Ledong (LD)	1311.1	24.7	18°25′15″N，108°53′40″E
5	Dongfang (DF)	949.7	25.3	18°48′34″N，108°39′35″E

Table S2. Physicochemical properties of *C. equisetifolia* forest soils along a precipitation gradient, Hainan Island.

Sample classification	Sample	WC	ST	pH	OM	OC	TN	HN	AP
Upper Soil	WNS	0.0425±0.0019i	28.61±0.15b	6±0.27e	15.58±1.3a	9.04±0.76a	0.64±0.1bc	43.41±1.09a	14.31±2.07b
	DZS	0.0705±0.0019d	27.86±0.12e	8.41±0.08c	3.65±0.28ef	2.12±0.16ef	0.65±0.19bc	15.14±7.87cd	2.01±0.2e
	CMS	0.0615±0.0019f	27.63±0.08f	7.24±0.02b	11.27±1.66b	6.54±0.96b	1.02±0.24a	36.76±7.49a	4.76±0.39d
	LDS	0.128±0.0024a	28.88±0.15a	6.44±0.18d	10.19±1.05bc	5.91±0.61bc	0.55±0.03bc	27.77±2.15b	6.66±1.34c
	DFS	0.0675±0.0019e	28.6±0.09b	6.92±0.04a	3.03±0.28ef	1.76±0.17ef	0.21±0.02e	8.9±4.2d	6.12±0.42c
Lower Soil	WNL	0.0465±0.0019h	28.11±0.12d	5.45±0.11f	9.21±1.85c	5.34±1.07c	0.44±0.03cd	24.42±4.1b	17.11±0.88a
	DZL	0.0695±0.0019de	27.85±0.08e	7.32±0.15b	2.22±0.27f	1.29±0.16f	0.7±0.02bc	10.53±5.96cd	1.86±0.29e
	CML	0.0875±0.0019c	28.33±0.08c	7.35±0.02b	3.7±0.39ef	2.15±0.23ef	0.43±0.06cd	16.94±2.41c	2.32±0.18e
	LDL	0.1065±0.0019b	28.87±0.12a	6.61±0.22d	6.1±0.54d	3.54±0.31d	0.33±0.03de	15.76±2.38cd	4.34±0.62d
	DFL	0.0515±0.0019g	28.54±0.09b	7.37±0.04b	2.44±0.1ef	1.42±0.06ef	0.19±0.02e	10.77±6.92cd	3.79±0.3d

Table S3. Microbial biomass characteristics of *C. equisetifolia* forest soils along a precipitation gradient.

Sample classification	Sample	MBC	MBN	MBP
Upper Soil	WNS	0.085±0.043c	0.022±0.009a	0.00039±0.00035a
	DZS	0.099±0.058bc	0.015±0.009a	0.00041±0.0004a
	CMS	0.18±0.042a	0.014±0.012a	0.0005±0.00031a
	LDS	0.133±0.051abc	0.014±0.009a	0.00059±0.00044a
	DFS	0.079±0.014c	0.022±0.016a	0.0005±0.00035a
Lower Soil	WNL	0.091±0.022c	0.015±0.006a	0.0008±0.00035a
	DZL	0.128±0.034abc	0.016±0.006a	0.00042±0.00031a
	CML	0.155±0.058abc	0.029±0.009a	0.0003±0.00024a
	LDL	0.11±0.053bc	0.02±0.013a	0.00053±0.00031a
	DFL	0.098±0.041bc	0.026±0.009a	0.00067±0.00011a

1. Explanation of sample codes: Codes are listed in order of decreasing mean annual precipitation. The first two letters indicate the site: WN (Wanning), DZ (Danzhou), CM (Chengmai), LD (Ledong), DF (Dongfang), all located in Hainan Province, China. The final letter indicates the soil layer: S for upper soil, L for lower soil.

2. Abbreviations:

MAP	Mean Annual Precipitation
MAAT	Mean Annual Air Temperature
LT	Litter Thickness
WC	Soil Water Content
ST	Soil Temperature
pH	Soil pH
OM	Soil Organic Matter
OC	Soil Organic Carbon
TN	Total Nitrogen
HN	Hydrolyzable Nitrogen
AP	Available Phosphorus
MBC	Microbial Biomass Carbon
MBN	Microbial Biomass Nitrogen
MBP	Microbial Biomass Phosphorus

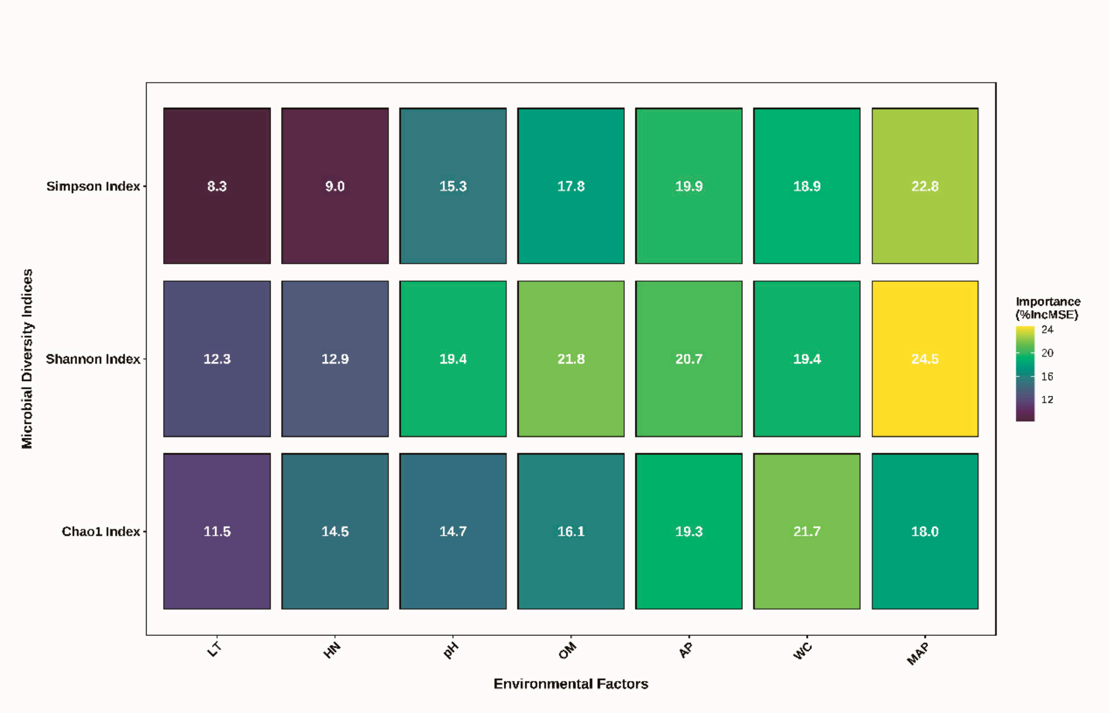

Figure S1. Variable importance plot from Random Forest analysis, ranking environmental factors by their contribution to variations in soil microbial diversity indices (Chao, Shannon, and Simpson indices).

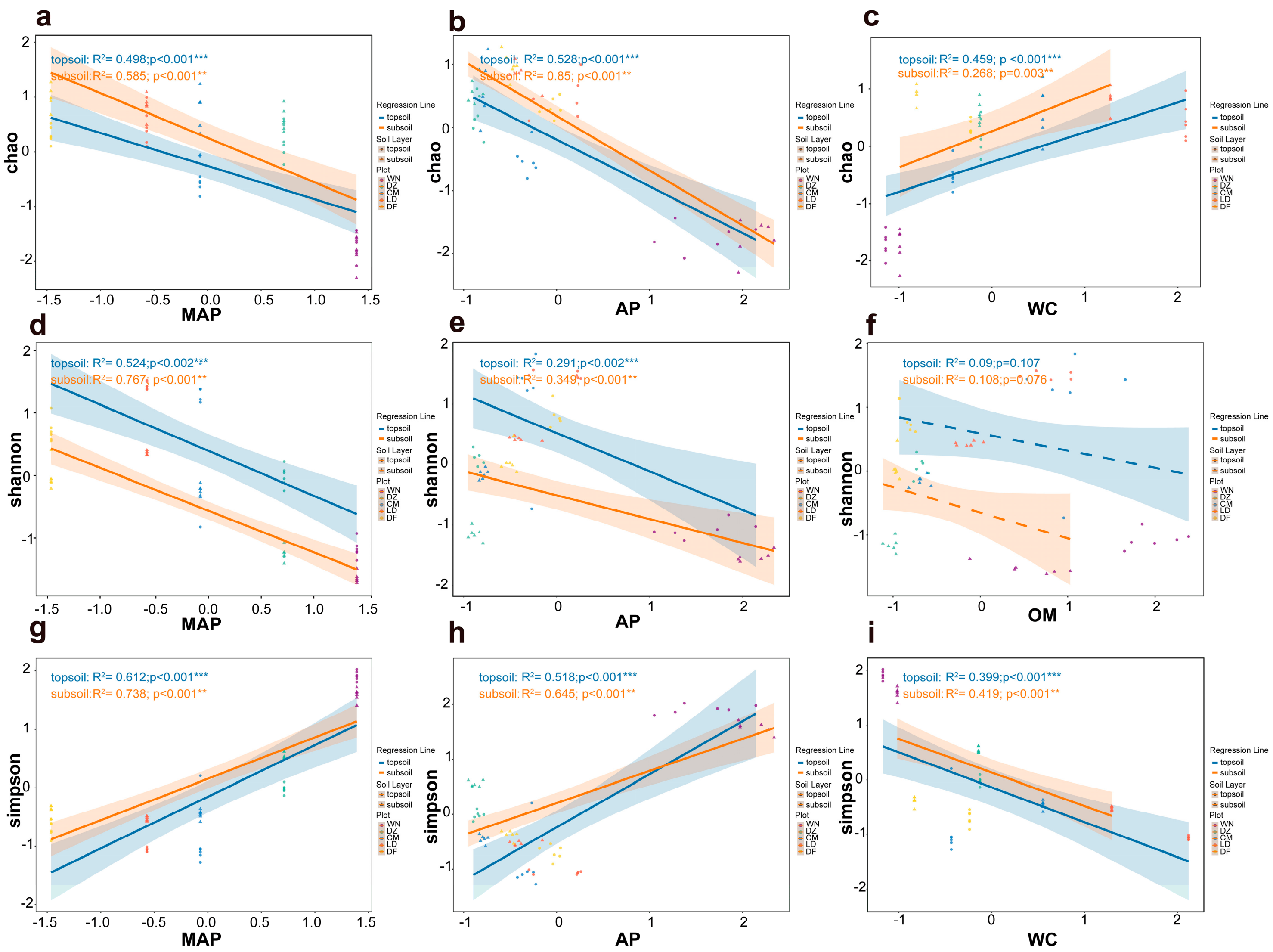

a
topsoil: R^2= 0.498;p<0.001***
subsoil:R^2= 0.585; p<0.001**
chao
MAP
b
topsoil: R^2= 0.528;p<0.001***
subsoil: R^2= 0.85; p<0.001**
chao
AP
c
topsoil: R^2= 0.459; p <0.001***
subsoil:R^2= 0.268; p=0.003**
chao
WC
d
topsoil: R^2= 0.524;p<0.002***
subsoil:R^2= 0.767; p<0.001**
shannon
MAP
e
topsoil: R^2= 0.291;p<0.002***
subsoil:R^2= 0.349; p<0.001**
shannon
AP
f
topsoil: R^2= 0.09;p=0.107
subsoil: R^2= 0.108;p=0.076
shannon
OM
g
topsoil: R^2= 0.612;p<0.001***
subsoil:R^2= 0.738; p<0.001**
simpson
MAP
h
topsoil: R^2= 0.518;p<0.001***
subsoil:R^2= 0.645; p<0.001**
simpson
AP
i
topsoil: R^2= 0.399;p<0.001***
subsoil:R^2= 0.419; p<0.001**
simpson
WC
Regression Line
topsoil
subsoil
Soil Layer
topsoil
subsoil
Plot
WN
DZ
CM
LD
DF

Figure S2. Correlation between environmental factors and soil microbial diversity indices in *C. equisetifolia* forest soils along a precipitation gradient.

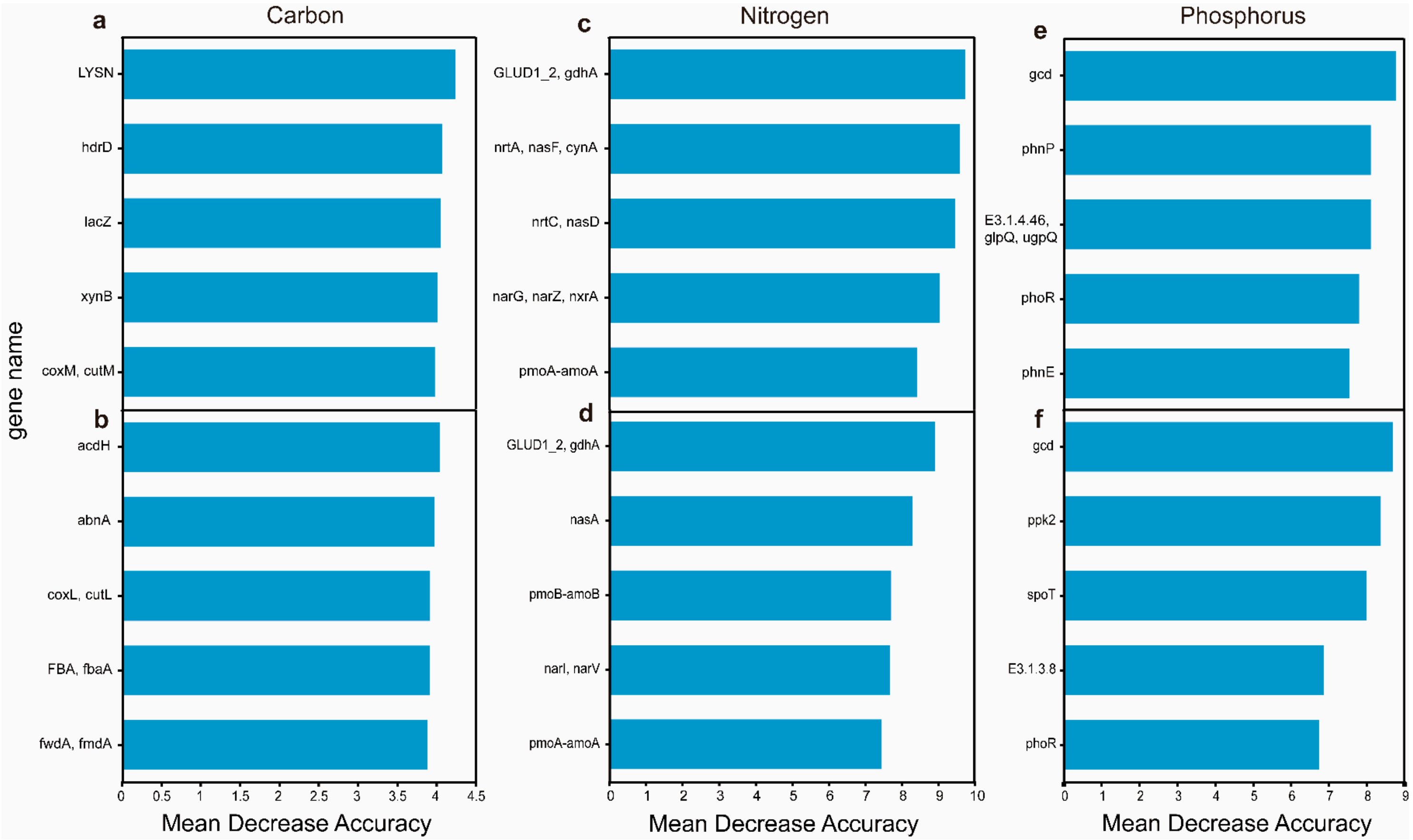

Figure S3. Ranking of key functional genes (KEGG name level) by their relative importance in *C. equisetifolia* forest soils along a precipitation gradient.
